# Structure of the Mon1-Ccz1 complex reveals molecular basis of membrane binding for Rab7 activation

**DOI:** 10.1073/pnas.2121494119

**Published:** 2022-02-01

**Authors:** Björn U. Klink, Eric Herrmann, Claudia Antoni, Lars Langemeyer, Stephan Kiontke, Christos Gatsogiannis, Christian Ungermann, Stefan Raunser, Daniel Kümmel

**Affiliations:** ^a^Department of Structural Biochemistry, Max Planck Institute of Molecular Physiology, 44227 Dortmund, Germany;; ^b^Institute of Biochemistry, University of Münster, 48149 Münster, Germany;; ^c^Department of Biology/Chemistry, Osnabrück University, 49076 Osnabrück, Germany;; ^d^Center of Cellular Nanoanalytics, Osnabrück University, 49076 Osnabrück, Germany

**Keywords:** GTPase, GEF, trafficking, endosome, cryo-EM

## Abstract

Activation of the GTPase Rab7/Ypt7 by its cognate guanine nucleotide exchange factor (GEF) Mon1-Ccz1 marks organelles such as endosomes and autophagosomes for fusion with lysosomes/vacuoles and degradation of their content. Here, we present a high-resolution cryogenic electron microscopy structure of the Mon1-Ccz1 complex that reveals its architecture in atomic detail. Mon1 and Ccz1 are arranged side by side in a pseudo-twofold symmetrical heterodimer. The three Longin domains of each Mon1 and Ccz1 are triangularly arranged, providing a strong scaffold for the catalytic center of the GEF. At the opposite side of the Ypt7-binding site, a positively charged and relatively flat patch stretches the Longin domains 2/3 of Mon1 and functions as a phosphatidylinositol phosphate–binding site, explaining how the GEF is targeted to membranes. Our work provides molecular insight into the mechanisms of endosomal Rab activation and serves as a blueprint for understanding the function of members of the Tri Longin domain Rab-GEF family.

## Significance

Rab GTPases are central regulators of intracellular trafficking and serve as markers of organelle identity. They act as molecular switches, and their activation requires precise spatiotemporal control. Members of the family of the Tri Longin domain (TLD) Rab-GEFs (guanine nucleotide exchange factors) act as activators of a subset of Rabs that play a critical role in late endosomal biogenesis. Genetic defects associated with TLD Rab-GEFs cause developmental diseases, but the underlying mechanisms are only partly understood. The determination of the structure of the TLD Rab-GEF Mon1-Ccz1 presented here provides a molecular basis for understanding the function and regulation of these proteins.

Rab GTPases are molecular switches that function as markers of organelle identity and coordinate intracellular trafficking as part of the conserved fusion machinery (1). The cycling of Rab GTPases between the inactive GDP-bound and the active GTP-bound form is tightly controlled by GTPase-activating proteins (GAPs) and guanine nucleotide exchange factors (GEFs) (2). While GAPs promote the intrinsically low GTP hydrolysis rate of Rabs to switch them off, GEFs stimulate nucleotide release and the loading of Rab with GTP to convert the GTPase to its active conformation. Inactive Rabs are kept cytosolic by the GDP dissociation inhibitor (GDI), which binds the Rab prenyl anchor (3). GDI can be removed from Rab GTPases by the GDI displacement factor (4), and the exchange of GDP with GTP also couples the association of Rab GTPases with membranes. Thus, the spatiotemporal regulation of Rab GTPases and downstream fusion events ultimately depend on the activation of their cognate GEFs.

Tri Longin domain (TLD) Rab-GEFs comprise one of many GEF families known. They contain at least two subunits, each of which is predicted to consist of three Longin domains (LDs) (5, 6). The TLD GEF family comprises the universally conserved Rab7-GEF Mon1-Ccz1 (MC1) (7–9) and two other complexes specific to metazoans, namely BLOC-3 (biogenesis of lysosome-related organelles complex-3, which includes Hps1 and Hps4) and Inturned-Fuzzy. BLOC-3 is the GEF for Rab32 and Rab38 on lysosome-related organelles (10), and mutations in BLOC-3 cause the genetic disease Hermansky–Pudlak syndrome (11). Inturned-Fuzzy, which acts as the GEF of Rab23 (5), has been implicated in the establishment of planar cell polarity and ciliogenesis and was described as part of the planar cell polarity effector complex in flies and the CPLANE (ciliogenesis and planar cell polarity effector) complex in mammalian cells (12). Several studies showed that in metazoans, TLD RabGEFs do not consist of two subunits but have additional non-TLD proteins bound to the heterodimeric core (13–17). The function of these additional subunits in the “enlarged” TLD RabGEF complexes is currently not clear.

The best-studied TLD Rab-GEF is MC1, which activates Rab7 (Ypt7 in yeast) in endosomal maturation and autophagy. In yeast, it has been demonstrated that MC1-dependent recruitment of Ypt7 to both late endosomes/multivesicular bodies and autophagosomes is required for the fusion of these organelles with the vacuole and degradation of the respective cargo (7, 18). This process is conserved in plants and mammalian cells (9, 14, 19). The metazoan MC1 complex contains a third subunit, namely Bulli/RMC1; however, the function of this protein remains elusive (14–16). Importantly, Bulli/RMC1 is not required for Rab5-dependent Rab7 activation and is thus not involved in regulating the GEF activity of MC1 (20). Recently, the uncharacterized protein C5orf51 was identified as an interactor of MC1 that links the GEF complex to mitophagy (17), yet the underlying mechanism remains to be determined.

We have previously identified the structure of Ypt7 bound to the MC1 core, which comprises a heterodimeric complex of the first Longin domains of Mon1 and Ccz1, respectively (21). The structure revealed a mechanism that involves remodeling of the GTPase switch regions. In the GEF-bound conformation, MC1 binding opens the nucleotide-binding pocket of Ypt7, which directs a lysine residue of Ypt7 into the Mg^2+^-binding pocket, thus favoring displacement of the bound nucleotide.

Although the MC1 core is required and sufficient for the GEF activity of the complex (21), functional studies in yeast showed that the catalytic core complex was unable to rescue the vacuolar fragmentation phenotype of *mon1*Δ or *ccz1*Δ strains and did not properly localize in cells. Thus, LD2 and LD3 as well as the rest of the complex are likely involved in correct recruitment of MC1 to the proper organelle membrane. Previous studies have identified GTPases of the Rab5 family and phosphatidylinositol phosphate (PIP) lipids as recruiting factors that promote binding of MC1 to endosomal membranes (20, 22, 23). On autophagosomes, Atg8 supports Mon1-Ccz1 function (16, 18). However, the mechanistic basis underlying these processes remains unclear.

To gain molecular insight into the targeting mechanism of MC1 and to understand how the complex is built in three dimensions, we determined the cryogenic electron microscopy (cryo-EM) structure of a stable MC1 complex from *Chaetomium thermophilum*. We observe a unique arrangement of the three LDs of each subunit within the complex and identify a conserved basic surface on MC1 that defines the orientation of the complex on lipid bilayers. Based on this, we developed a model of the function of Mon1-Ccz1 on membranes. The structure of Mon1-Ccz1 thus provides a blueprint for the architecture and function of the TLD RabGEF family.

## Results

### Cryo-EM Structure of CtMC1Δ.

For our studies of MC1, we used the complex from the thermophilic fungus *C. thermophilum* (CtMC1), which has the same domain organization as the well-studied *Saccharomyces cerevisiae* ScMC1 but proved to be more stable and easier to handle in vitro (*SI Appendix*, Figs. S1*A* and S2*A*). Although we could express full-length CtMC1 and purify the complex to homogeneity (*SI Appendix*, Fig. S3*A*), the single particles were not randomly oriented on the cryo-EM grids, which prevented us from obtaining a cryo-EM structure. Since different grid preparation methods did not help, we modified the protein by the deletion of predicted disordered regions to change its possible orientation on the cryo-EM grid.

To ensure that the deletions do not affect the functionality of the complex, we first tested the respective constructs of ScMC1 in yeast cells. ScMon1 and ScCcz1 both are required for maintaining vacuolar morphology and localize as punctate structures in late endosomal dots next to the vacuole. A ScCcz1 construct lacking a predicted 133-amino-acid loop in LD2 (residues 270 to 403) still localized in perivacuolar punctate structures and complemented a *ccz1*Δ strain to restore vacuolar morphology (*SI Appendix*, Fig. S3*B*), showing that endosomal maturation was functional. The deletion of the first 158 amino acids of ScMon1, comprising an N-terminal predicted disordered domain, rescued vacuole morphology in *mon1*Δ cells, but endosomal targeting of ScMon1Δ158 was reduced (*SI Appendix*, Fig. S3*C*). However, ScMon1 only lacking amino acids 1 to 140 was functional and showed localization comparable to wild type (*SI Appendix*, Fig. S3*C*), indicating that part of the Mon1 disordered domain adjacent to LD1 has an important role in subcellular targeting of MC1.

**Fig. 1.**
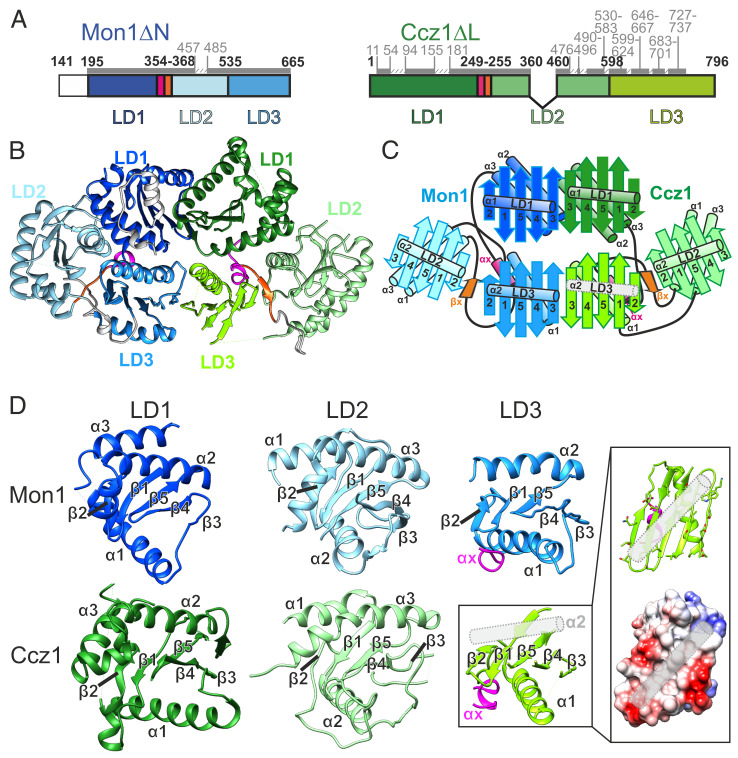
Cryo-EM structure of CtMC1Δ. (*A*) Schematic representation of the constructs comprising CtMC1Δ. Construct and domain boundaries are labeled in bold, and the modeled regions are marked by dark gray bars and light gray labels. (*B*) Atomic model of the Mon1- (blue) Ccz1 (green) complex. (*C*) Domain architecture and relative arrangement of the six Longin domains in the MC1 complex. LD1 of both protomers are canonical Longin domains (ββαβββαα), LD2 are of the roadblock Longin type (αββαβββα), and LD3 resemble the lamtor Longin type (ββαβββα), which is completed by a helix α_X_ (magenta) originating from the linker region between LD1 and LD2. We did not observe density for the second helix of the Ccz1 LD3 (gray rod). (*D*) Closeup view of the LD domains in similar orientations. For the LD3 domain of Ccz1, a coulomb surface potential of the expected binding site of α_2_ is shown. The exposed surface of LD3 seems favorable to accommodate a helix, and the probable position of α_2_ is shown as gray rod.

We then produced an equivalently truncated complex CtMC1Δ (CtMon1 141 to 665, CtMon1ΔN; CtCcz1 1 to 796Δ361 to 460, CtCcz1ΔL; Fig. 1*A*), which expressed at an improved yield and also exhibited higher stability than the wild type (*SI Appendix*, Fig. S3*D*). Importantly, CtMC1Δ had the same catalytic GEF activity (*SI Appendix*, Fig. S3 *E*–*G*).

The orientation of the CtMC1Δ particles on the cryo-EM grids was tremendously improved in comparison to the wild type, allowing us to obtain a three-dimensional (3D) reconstruction of the complex with an average resolution of 3.85 Å (*SI Appendix*, Table S1 and Figs. S4 and S5). The quality of the map was sufficient to build an atomic model of ∼75% of CtMC1Δ, including all Longin domains of Mon1 and Ccz1 (Fig. 1 *B* and *C* and *SI Appendix*, Fig. S6). Several large, predicted loops were not visible in the map, likely due to high flexibility (*SI Appendix*, Figs. S1*B* and S2 *B*–*D*).

The three Longin domains (LDs) of both Mon1 and Ccz1 are triangularly arranged and form together the backbone of MC1, which has a pseudo twofold symmetry (Fig. 1 *B* and *C*). All LDs share the basic Longin fold, comprising a central five-stranded antiparallel β-sheet with conserved topology (β_2_-β_1_-β_5_-β_4_-β_3_), two α-helices on one side of the sheet (α_1_ and α_3_), and a single α-helix on the opposite side of the sheet (α_3_) (Fig. 1*D*). A pairwise comparison of LD1s, LD2s, and LD3s shows that the structures of the LDs of Mon1 and Ccz1 are highly similar. Helix α_2_ of LD3 in Ccz1 could not be modeled because of the lack of clear density. However, the surface properties of Ccz1-LD3 at this region are very hydrophobic, and we expect that α_2_ occupies this area (Fig. 1*D*). While the LD1s and LD2s are classical Longin-type and roadblock domains, respectively (24), LD3 is a lamtor-type domain in both Mon1 and Ccz1 (Fig. 1*D*). Lamtor-type domains lack one helix for completion of the longin fold, which can be contributed by another protein (25). In Mon1 and Ccz1, this function is fulfilled by an additional helix, which we termed α_X_, inserted between LD1 and LD2 (*SI Appendix*, Figs. S1*B* and S2*B*).

Mon1 and Ccz1 interact via their LD1 and LD3 domains. The respective LDs (LD1-LD1 and LD3-LD3) share a large interface and form two continuous cross-subunit β-sheets (Fig. 1 *B* and *C*). This stabilizes the complex and provides a strong scaffold for the catalytic center that resides between the LD1s of Mon1 and Ccz1. The cross-subunit β-sheet formed between the LD3s even extends to the peripheral LD2s on both sides of the complex, forming an extra-long continuous β-sheet with 22 strands. The two continuous β-sheets are a defining structural feature of MC1 that results in a two-layered complex architecture. While the upper layer, comprising the LD1 heterodimer, represents the subcomplex that is required and sufficient for the catalytic activity of MC1, the lower layer is necessary for the proper localization in cells (21). Thus, structurally and functionally, the MC1 complex can be divided into a top “catalytic” layer and a base “localization” layer.

**Fig. 2.**
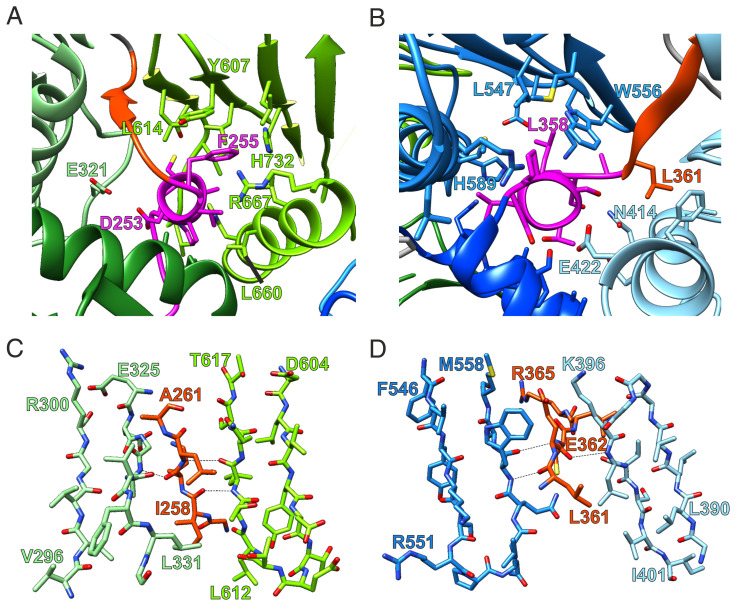
Molecular details of the CtMC1Δ structure. (*A* and *B*) Stabilization of the arrangement of three LD domains of Ccz1 (*A*) and Mon1 (*B*) by the short helix (α_X_, magenta), which by primary sequence is located in the linker region between LD1 and LD2 of each protomer. (*C* and *D*) The linker region after α_X_ continues as a β-strand (β_X_, orange ribbon), which passes through and augments the continuous β-sheets of LD2 and LD3.

The linker between LD1 and LD2, which contains helix α_X_ and an additional β-strand (β_X_), plays a central role in the interaction between the LDs of Mon1 and Ccz1, respectively. While α_X_ swaps to LD3 and interacts with helix α1 (Fig. 2 *A* and *B*), β_X_ completes the continuous β-sheet between LD3 and LD2 (Fig. 2 *C* and *D* and *SI Appendix*, Figs. S2*B* and S3*B*). Thus, this linker acts as a central connector and stabilizer of the three longin domains of Mon1 and Ccz1. Secondary structure predictions show that the α_X_-β_X_ motif between LD1 and LD2 is not only conserved in Mon1 and Ccz1 homologs of other species (*SI Appendix*, Figs. S1*B* and S2*B*) but also in all TLD Rab-GEFs of metazoans (CPLANE, BLOC-3). It thus represents a unique and defining structural feature of TLD Rab-GEFs.

### Assembly of the Active Mon1-Ccz1 Complex on Membranes.

**Fig. 3.**
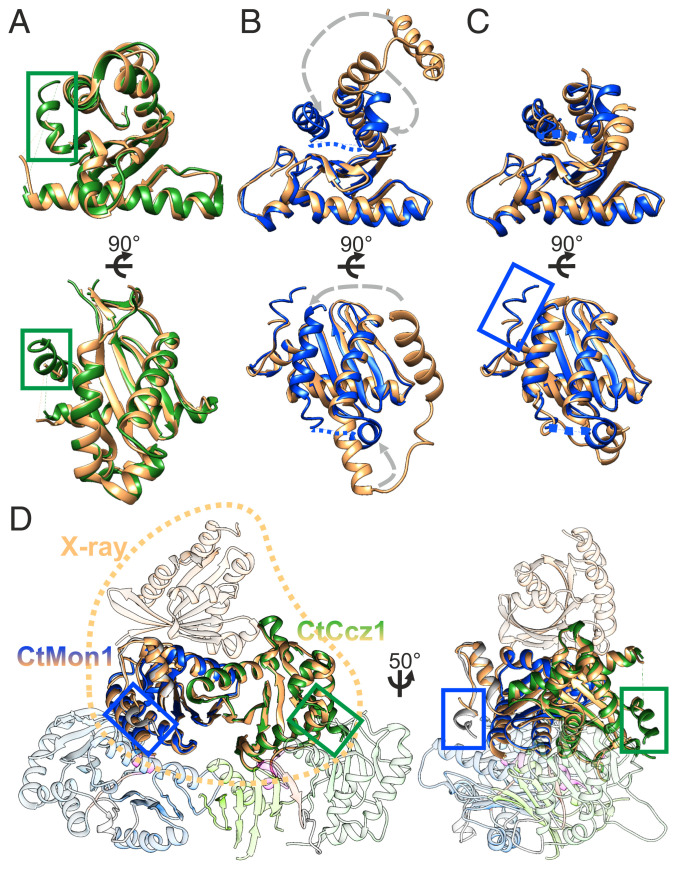
Comparison of the CtMC1Δ cryo-EM with the Mon1-LD1/Ccz1-LD1/Ypt7 crystal structure. (*A*) The LD1 domains of Ccz1 agree with an rmsd of 0.76 Å over 157 residues. In the cryo-EM structure, residues A44-H54 form a helix (green box) because of stabilizing interactions with LD2, which was not resolved in the crystal structure. (*B*) The LD1 domains of Mon1 match poorly (rmsd of 0.84 Å over 94 residues) because of a domain swap of the helical region L314-S353 in the crystal structure. (*C*) A domain swap–corrected model of the Mon1-LD1 crystal structure (21) matches with an rmsd of 0.95 Å over 124 residues. In addition, residues M195-E201, which are not visible in the crystal structure, form a short helical segment (α_-1_, blue box) that interacts with helix α1 of Mon1-LD3. (*D*) Overlay of the CtMC1Δ complex with the crystallized LD1 domains of Mon1 and Ccz1 bound to Ypt7 (PDB identification: 5LDD) shows a highly similar arrangement of the LD1 domains in the presence and absence of LD2 and LD3 (rmsd 0.766 Å over 252 residues), indicating that the full complex can bind to Ypt7 in the same way as the individual LD1 domains.

To identify if there are conformational differences between the cryo-EM structure of CtMC1Δ and the crystal structure of Mon1-LD1/Ccz1-LD1/Ypt7 (Protein Data Bank [PDB] identification: 5LDD) (21), we overlaid and compared these two structures (Fig. 3). Mon1 contains two additional helices (residues 198 to 201 and 210 to 220) that are not part of the longin domains. These helices α_0_ and α_-1_ (*SI Appendix*, Fig. S1*B*) stabilize LD1 and its intramolecular interaction with LD3, respectively. Similarly, the cryo-EM structure reveals an additional helical segment in Ccz1-LD1 (residues 44 to 54), which was not observed in the crystal structure (21). This helix participates in formation of the Ccz1 LD1-LD2 interface, which likely results in a mutual stabilizing effect (Fig. 3*A*). Expectedly, a domain swap of Mon1 that occurred during crystallization is not relevant in the context of the full complex (Fig. 3 *B* and *C*). The cryo-EM structure and domain swap–corrected model derived from the X-ray structure are virtually identical, with an rmsd of 0.766 Å over 252 of 280 Cα atoms (Fig. 3*D*). This shows that the presence of the LD2 and LD3 domains does not change the conformation of LD1, suggesting that the full complex binds to Ypt7 in the same way as the individual LD1 domains in the crystal structure.

**Fig. 4.**
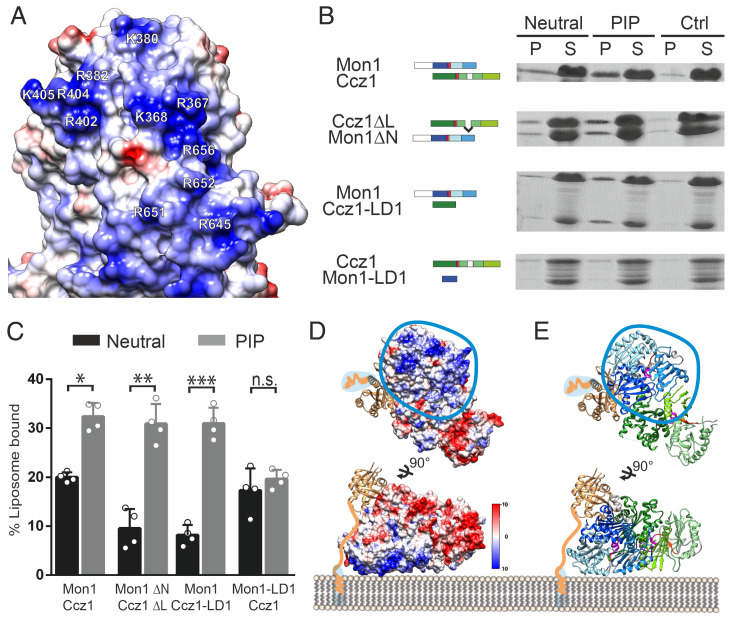
Lipid binding of MC1 and model for membrane association of the MC1-Ypt7 complex. (*A*) Coulomb surface potential of the basic interface on Mon1 LD2 and LD3 that is defined by multiple positively charged residues. Key residues clustered in primary and tertiary structure and conserved in Mon1 homologs are labeled. (*B*) Sedimentation assays with different CtMon1 and CtCcz1 constructs. Experiments were carried out with liposomes generated with a neutral or PIP-containing mix or without liposomes (Ctrl). Pellet fractions containing the liposomes (P) and supernatants (S) were analyzed by sodium dodecyl sulphate–polyacrylamide gel electrophoresis and Coomassie staining. (*C*) Quantification of *B* from *n* = 4 independent experiments. Data are presented as mean ± SD, and the significance was calculated using a Student’s *t* test (*P < 0.05, **P < 0.001, ***P < 0.0001, n.s. not significant). (*D* and *E*) Model of the Mon1-Ccz1 complex with prenylated Ypt7 binding to membranes. The probable binding interface is defined by the large basic patch located on Mon1. The prenylated C terminus of Ypt7 is connected to the G domain by a flexible linker that allows binding of the core Ypt7 domain to MC1 4 to 5 nm above the membrane. (*D*) Coulomb surface potential (42). (*E*) Cartoon representation.

An electrostatic surface potential map of CtMC1Δ reveals a large positively charged patch stretching over LD2 and LD3 of Mon1 and a negatively charged region on the surface of LD1 and LD3 of Ccz1 (Fig. 4*A* and *SI Appendix*, Fig. S6*C*). The other regions of CtMC1 show an even distribution of charge and hydrophobicity (*SI Appendix*, Fig. S6 *C* and *D*). Since basic interfaces on peripheral membrane proteins are known to interact with negatively charged head groups of phospholipids via electrostatic interactions (26), the large positively charged surface on MC1 is likely the region where the complex resides on the membrane. In line with this, it was previously shown that the C-terminal domains of Mon1 and Ccz1 (LD2 and LD3) are required for proper localization of ScMC1 in yeast cells (21). In addition, this region of MC1 is flat, which is optimal for interacting with the membrane, and most of the involved basic residues are preserved in other Mon1 homologs (*SI Appendix*, Fig. S1*B*), suggesting that this interface is a conserved feature of the complex.

ScMC1 has been shown to interact with PIP lipids (20, 22, 23), which are known to interact with positively charged patches of proteins (27). Thus, PIP lipids could be the components in late endosomes/multivesicular bodies and autophagosomes that interact with MC1. To find out whether this is true, we first tested if the PIP binding is conserved in CtMC1 and found that just as ScMC1, CtMC1 bound to PIP-containing liposomes in a liposome sedimentation assay (Fig. 4 *B* and *C*). PIP binding was also observed for the truncated CtMC1Δ complex. We then tried to express and purify single LD2, LD3, or LD2/3 of either Mon1 or Ccz1. However, this was not successful. To perform a mapping of the PIP-binding domain, we instead produced subcomplexes of CtMC1 that lack LD2/3 and were stable as recombinant protein. A CtMC1 subcomplex containing full-length Mon1 and Ccz1-LD1, but lacking Ccz1-LD2/3, still bound to PIP liposomes. In contrast, full-length Ccz1 bound to Mon1-LD1 did not show PIP interaction (Fig. 4 *B* and *C*). Thus, LD2/3 of Mon1 mediate the PIP-dependent membrane binding of MC1. Taken together, we conclude that the basic patch formed by LD2/3 of Mon1 is responsible for binding to PIP lipids.

This result elucidates how MC1 is oriented on the membrane surface (Fig. 4 *D* and *E*). When the basic patch lies flat on the membrane, the entire base layer of the MC1 complex is positioned at the bilayer interface. The catalytic top layer, in contrast, is located at the opposite side with the LD1s that form the catalytic site facing outwards toward the cytosol. This will position Ypt7 on top of the complex, ∼40 Å away from the membrane surface. Ypt7 is anchored in the membrane by a prenyl modification at the C terminus. The prenylation site is linked to the G domain via a flexible hypervariable region of 27 amino acid length. As a peptide in an extended conformation may span ∼3 Å per peptide bond, Ypt7 can easily accommodate the proposed distance from the membrane. This model is also consistent with experimental data obtained by graphene-induced energy transfer measurements (28). Here, Ypt7 was observed 38 Å away from the membrane surface in the presence of Mon1-Ccz1.

## Discussion

The structure of MC1 reveals that Mon1 and Ccz1 each consist of three LDs, which interact by β-sheet complementation via both LD1s and LD3s. LD2 of Mon1 and Ccz1 flank the complex at either end. The catalytic core resides between the LD1s. We identify a basic patch on one face of the MC1 complex formed by LD2 and LD3 of Mon1 and show that these LDs are responsible for binding to PIP-lipid–containing membranes. The interaction via this patch orients the complex on the membrane such that the catalytic site is placed away from the membrane.

Interestingly, the arrangement of the MC1-Ypt7 complex on the membrane is strongly reminiscent of how Rab1 and its cognate GEF, the TRAPPIII complex, may associate with the membrane (29, 30). Charge interactions of TRAPP with phospholipid head groups position the complex such that the active site of TRAPP also faces away from the lipid bilayer, and Rab1 binds on top. Here, a steric gating mechanism was identified that controls access of different GTPases through the length of their hypervariable region, which will prohibit interaction with the active site if too short (31). The TRAPPIII complex also directly interacts with the hypervariable domain. These mechanisms are important to improve the specificity of TRAPP in different cellular contexts because this GEF can accept several Rab GTPases as substrate. In contrast, MC1 is rather specific for Ypt7 and may not demand further control of fidelity (5, 21). However, an interaction with the hypervariable region of Ypt7 could improve the affinity and activity of MC1.

A general advantage of the outward-facing orientation of the GEF active site could be to facilitate access to the Rab GTPase substrate. Rab GTPases, in contrast to members of other small GTPase families, are bound to a GDI (guanine nucleotide dissociation inhibitor) chaperone in the cytosol in their inactive, GDP-bound conformation. Efficient Rab activation thus requires displacement from the GDI, insertion of the prenyl anchor into the membrane, and nucleotide exchange, which may occur in a sequential order or concurrently. It is conceivable that this process is catalyzed more effectively when the active site of the GEF is oriented toward the cytosol from which the substrate approaches.

Liposome sedimentation studies in vitro demonstrated that MC1 does not show any specificity for anionic lipids (20). The complex binds with the same efficiency to membranes containing PIPs, PIP_2_s, or phosphatidylserine when lipid concentrations are adjusted for the respective charge of the head groups, indicating that binding is driven by unspecific electrostatic charge interactions in agreement with previous findings (22). This is consistent with our observation that Mon1 LD2 and LD3, which are responsible for membrane binding, form an extensive basic patch but no obvious binding pocket that could specifically recognize a particular lipid head group. Whether and how Ccz1 contributes to membrane binding is not yet known.

The interaction of MC1 with anionic lipids likely increases the affinity of membrane binding of the complex and can dictate the orientation of MC1 on the membrane. The requirement of PI3P for endosomal maturation and MC1 function may result from the fact that it is the predominant anionic lipid present on endosomes (32). For several endosomal proteins, PI3P-specific binding domains have been described that result in targeting to endosomes, for example, the FYVE (Fab1/YOTB/Vac1/EEA1) domain containing endosomal tethering protein EEA1 (Early Endosome Antigen 1) (33). In contrast, biochemical and structural data for MC1 demonstrate that it cannot discriminate between different PIPs. We therefore conclude that PI3P is unlikely to serve as a specific localization cue for MC1. Additional factors that promote MC1 membrane binding synergistically with PIPs, like Vps21/Rab5 on endosomes (20) and Atg8 on autophagosomes (18), will contribute to specificity. In this context, it is interesting to note that the predicted disordered N-terminal domain of Mon1 is required for localization of MC1 to endosomes. Even though not resolved in our structure, this part of the protein stabilizes Mon1. It is plausible that this sequence could represent a regulatory element to bind other factors on the membrane. In particular, Rab5 proteins can bind Mon1, and this interaction is regulated through phosphorylation by the casein kinase Yck3 (20, 23). Our results support a model that the flexible N-terminal domain of Mon1 either directly binds Rab5 or regulates this interaction.

The Mon1-Ccz1 heterodimer has a unique architecture that, to our knowledge, has not been observed in other complexes before. The structure of the subunits and of the assembled complex can serve as a blueprint for understanding the structural organization and function of other TLD RabGEFs, which have not been characterized structurally thus far. Our findings enable further studies to investigate how these complexes are specifically recruited by protein and lipid interactions to their respective target organelle. Based on the structure presented here, it will also be important to determine how additional subunits bind to form enlarged TLD Rab-GEF complexes and influence functionality.

## Materials and Methods

CtMon1 and CtCcz1 were coexpressed in BL21 *Escherichia coli* with N-terminal GST- and 6xHis-SUMO tags, respectively, and purified via affinity chromatography followed by proteolytic tag removal and size exclusion chromatography. For vitrification, 3 µl MC1-Ccz1 complex at 0.86 mg ⋅ ml^−1^ concentration was applied to a glow-discharged Quantifoil 2/1 holey carbon grid (Quantifoil), blotted for 3 s, and plunged in liquid ethane using a Vitrobot (FEI). Cryo-EM datasets were collected on a Titan Krios electron microscope (FEI) equipped with a post-column energy filter, a Volta phase plate (34), and a field emission gun operated at 300 kV acceleration voltage. Three datasets were individually processed using crYOLO (35) and SPHIRE (36). Per-particle contrast transfer function correction followed by 3D classification and postprocessing was performed in Relion (37). The final 3D reconstruction has an average resolution of 3.85 Å as estimated by the “gold standard” criterion of Fourier shell correlation = 0.143 between two independently refined half maps. To facilitate map interpretation, we used maps for model building, which were sharpened by local anisotropic sharpening in Phenix (38) and by a deep-learning–based approach by DeepEMhancer (39) with the implemented highRes training model. For model building, a combination of de novo structure prediction by TRRosetta (40) and manual model building in Coot (41) was employed. The electron density map was deposited to the Electron Microscopy Data Bank (EMDB,accession code EMD-14066) and the final model to the PDB (accession code 7QLA).

For sedimentation assays, 400-nm extruded liposomes with 5% sucrose were used. Final concentrations of 0.5 mM lipids and 1 µM protein were incubated for 20 min at room temperature, the liposomes were pelleted, and the supernatant fraction was subjected to acetone precipitation. Samples were analyzed via sodium dodecyl sulphate–polyacrylamide gel electrophoresis and Coomassie staining, and band intensities were quantified with Bio-Rad Image Lab. GEF assays were performed with 2 µM CtYpt7-MANT-GDP and 0 to 2 µM CtMC1 complex essentially as described before (21). Yeast strains transformed with pRS406 plasmids carrying GFP-tagged versions of Mon1 or Ccz1 (*SI Appendix*, Table S3) were analyzed with a Delta Vision Elite (GE Heathcare) microscope as previously described (22).

## Supplementary Material

Supplementary File

## Data Availability

Protein structure and electron microscopy map data have been deposited in the PDB and EMDB (PDB-7QLA and EMD-14066, respectively).
