# Supplementary material for: Structure of the Mon1-Ccz1 complex reveals molecular basis of membrane binding for Rab7 activation
Source: Proc Natl Acad Sci U S A. 2022 Feb 1;119(6):e2121494119. doi: 10.1073/pnas.2121494119 (PMC8833172; doi:10.1073/pnas.2121494119)
Supplement: Supplementary File [file pnas.2121494119.sapp.pdf]

PNAS
www.pnas.org

Supplementary Information for
Structure of the Mon1-Ccz1 complex reveals molecular basis of membrane binding for Rab7 activation.

Björn U. Klink[1,a,b], Eric Herrmann[1,c], Claudia Antoni[a], Lars Langemeyer[d], Stephan Kiontke[d], Christos Gatsogiannis[a,b], Christian Ungermann[d,e], Stefan Raunser[a,*], Daniel Kümmel[c,d,*]

[a] Max-Planck-Institute of Molecular Physiology, Department of Structural Biochemistry, 44227 Dortmund, Germany

[b] Current address: University of Münster, Institute for Medical Physics and Biophysics and Center for Soft Nanoscience, 48149 Münster, Germany

[c] University of Münster, Institute of Biochemistry, 48149 Münster, Germany

[d] Osnabrück University, Department of Biology/Chemistry, 49076 Osnabrück, Germany

[e] Center of Cellular Nanoanalytics (CellNanOs), Osnabrück University, 49076 Osnabrück, Germany

[1] These authors contributed equally.

* Correspondence to Stefan Raunser and Daniel Kümmel

Email: stefan.raunser@mpi-dortmund.mpg.de, daniel.kuemmel@wwu.de

This PDF file includes:

Supplementary text
Figures S1 to S6
Tables S1 to S3
SI References

Supplementary Material and Methods

Protein expression and purification

BL21 *E. coli* cells were co-transformed with the expression plasmids pCDF6P-CtMon1 (with N-terminal GST-tag and PreScission protease cleavage site) and pET28HS-CtCcz1 (with N-terminal 6xHis-SUMO tag) (Table S2). The cells were grown in TB media to OD_{600} = 0.6. After incubation for 30 minutes on ice, the protein expression was induced by adding 0.25 mM isopropyl-b-D-thiogalactoside. After 16 h shaking at 16°C, the cells were harvested at 3,000 xg for 15 minutes at 4°C. Cell pellets were resuspended in buffer A1 (50 mM NaH_2PO_4, 500 mM NaCl, 1 mM $MgCl_2$, 5% (v/v) glycerol, pH 7.3) with protease inhibitor mix HP (Serva) and lysed with a microfluidizer (Microfluidics). After centrifugation at 39,000 x*g* for 30 minutes at 4°C, the supernatant was transferred to self-packed glutathione agarose columns. After several rounds of washing with buffer A1, the proteolytic cleavage of the tags was achieved by 2 h incubation with SUMO protease and subsequent incubation overnight with PreScission protease. The complex was eluted and concentrated with a 30 kDa molecular weight cutoff Amicon Ultra concentrator. To remove the proteases and monomeric Mon1, the eluate was loaded on size exclusion chromatography column (SEC650, Bio-rad) pre-equilibrated with buffer A2 (25 mM HEPES, 250mM NaCl, 1mM MgCl2, 0.5 mM TCEP, pH 7.3). For Sedimentation assays, different construct combinations were expressed and purified accordingly, except that tag cleavage was omitted and the proteins were eluted with Buffer A1 supplemented with 20 mM glutathione and 12 mM DTT instead.
CtYpt7 was expressed in BL21 *E. coli* cells with the expression plasmid pCDF6P-CtYpt7fl and purified as described for CtMC1 with the exception that only PreScission was added for proteolytic cleavage.

Sedimentation assays

Liposomes were generated from neutral (82 mol% 1-palmitoyl-2-oleoyl phosphatidylcholine [POPC], and 18 mol% 1-palmitoyl-2-oleoyl phosphatidylethanolamine [POPE]) or PIP containing (79 mol% POPC, 18 mol% POPE, 2 mol% dipalmitoylphosphatidylinositol-3-phosphate [PI-3-P], and 1 mol% dipalmitoylphosphatidylinositol-3–5-bisphosphate [PI-3,5-P2]) lipid mixtures. The lipids were dried for at least 1 h in a speedvac and dissolved in 1 ml of buffer A2S (25 mM HEPES, 250 mM NaCl, 1 mM $MgCl_2$, 5% sucrose, pH 7.3) to a final lipid concentration of 2 mM. The liposome suspension was freeze/thawed for 5 times in liquid nitrogen and at 56°C, respectively. Multilamellar lipid vesicles were extruded 13 times through a 400 nm membrane to generate liposomes.
To co-pellet the protein and the liposomes, final concentrations of 0.5 mM lipids and 1 µM protein were added in buffer A2 to a final volume of 200 µL. After 20 min of incubation at room temperature, the liposomes were pelleted at 20,000 x*g* for 20 min at 4°C. The soluble supernatant fraction was separated from the membrane fraction in the pellet and precipitated with ice-cold 100% acetone on ice. All samples were analyzed via SDS-PAGE and subsequent Coomassie staining. The gels were scanned and the band intensity was quantified with Bio-rad Image Lab. Significance analyses were performed by two-tailed heteroscedastic t-test statistics.

GEF assays

Purified CtYpt7 was loaded with MANT-GDP in the presence of 20 mM EDTA for 30 min at 30°C. After quenching the loading reaction with 25 mM $MgCl_2$, CtYpt7-MANT-GDP was purified via size exclusion chromatography in Buffer A2. To determine the GEF-activity, 2 µM of CtYpt7-MANT-GDP complex were incubated with 0 - 2 µM of CtMC1 complex. The nucleotide exchange reaction was started by adding 0.1 mM GTP and monitored by the decrease of the fluorescence signal at λ_{em} 450 nm (λ_{ex} 354 nm) in intervals of 30 s at 25°C. Data were fitted against a first-order exponential decay ($y=y_0 + A\cdot e^{-x/t}$) to determine $k_{obs}=t^{-1}$ (s^{-1}). k_{obs} was plotted against the concentration of CtMC1 and k_{cat}/K_M ($M^{-1}\ s^{-1}$) was determined as the slope of the linear fit y=A·x+B.

Sample vitrification for cryo-EM

The concentration of the MC1-Ccz1 complex was adjusted to 0.86 mg ml^{-1} and 3 µl of sample was immediately applied to a glow-discharged Quantifoil 2/1 holey carbon grids (Quantifoil). Excess sample was automatically blotted for three seconds, and the grid was then plunged in liquid ethane using a Vitrobot (FEI).

Electron microscopy and image processing

Due to the small particle size and the tendency to form the best ice only in very densely decorated grids, we pursued the use of a Volta phase plate (VPP)(1) for data collection to enhance image contrast and this way improve particle selection and alignment.

Cryo-EM datasets were collected on a Titan Krios electron microscope (FEI) equipped with a post-column energy filter, a Volta phase plate (VPP) and a field emission gun (FEG) operated at 300 kV acceleration voltage. A total of 11,916 micrographs were recorded in three separate sessions on a K2 direct electron detector (Gatan) with a calibrated pixel size of 1.09 Å. The energy filter was used for zero-loss filtration with an energy width of 20 eV. In total 60 frames (each 250 ms) were recorded, resulting in a total exposure time of 15 s and a total electron dose of 72.4 to 73.7 $e^{-}/Å^{2}$. Data was collected using the automated data collection software EPU (FEI), with a defocus range of −0.25 to −1.2 µm. The position of the VPP was changed every 30 to 100 images, resulting in phase shifts of 20–150 degrees in >95% of all micrographs. Beam-induced motion was corrected for by using Motioncor2(2) to align and sum the 60 frames in each micrograph movie and to calculate dose-weighted and unweighted full-dose images. Initial CTF parameters were estimated from the unweighted summed images and from micrograph movies utilizing the 'movie mode' option of CTFFIND4(3). For subsequent steps of data processing using the software package SPHIRE/EMAN2(4), dose-weighted full dose images were used to extract dose-weighted and drift-corrected particles with a final window size of 180 × 180 pixels.

The three datasets of the Mon1-Ccz1 complex were individually processed by a combination of manual and automated particle picking using crYOLO(5) and 2D sorting using the iterative stable alignment and clustering (ISAC) as implemented in SPHIRE. The selected particles were then merged and further 2D sorted to obtain a cleaned particle stack with 2,833,930 particles. An initial model for 3D refinement was generated from the ISAC 2D class averages using RVIPER from SPHIRE, and was used as input for the first 3D refinement using MERIDIEN (3D refinement in SPHIRE). The obtained 3D reconstruction of MERIDIEN was then sharpened and filtered to its nominal resolution, and used as input for subsequent 3D refinements. The obtained 3D reconstructions were difficult to interpret in the surface regions of the complex and indicated conformational heterogeneity. We therefore converted the particle stack to the Relion data format using SPHIRE2RELION from the SPHIRE suite, and performed per-particle CTF correction followed by 3D classification into four classes in Relion(6). We used the best 3D class with 911.674 particles for further 3D refinement and postprocessing in Relion. The final 3D reconstruction has an average resolution of 3.85 Å, as estimated by the 'gold standard' criterion of FSC = 0.143 between two independently refined half maps. To further facilitate map interpretation, we also considered maps for model building which were sharpened by local anisotropic sharpening in Phenix(7) and by a deep-learning based approach by DeepEMhancer(8) using the implemented highRes training model.

Model building

For building an atomic model of the Mon1-Ccz1 complex, we used a combination of de novo structure prediction by TRRosetta(9) and manual model building in Coot(10). We were not able to generate stable de novo predictions of the LD2 and LD3 of Ccz1, which therefore had to be built *ab initio*. Atom clashes were removed by energy minimization (with torsion and Ramachandran restraints turned on) using PHENIX real space refinement(7), followed by manual refinement in Coot. The validity of the model was verified based on side chain consistency with the map in most parts of the model and a very good agreement of the LD1 domains with the crystal structure of the isolated Mon1/Ccz1 LD1 domains with Ypt7(11). In addition, the domain architecture and observed secondary structure elements in our final model match the domain architecture that was predicted based on sequence comparisons(12, 13) and the AlphaFold2 predictions (Jumper et al., 2021). The CtMC1 complex contains a large number of unresolved loops, which results in β-strands without connection to other structural elements in our 3D reconstruction. We therefore cannot exclude model imperfections particularly in the LD3 domain of Ccz1.

Visualization

Visualization, analysis and figure preparation was done with Chimera (UCSF)(14). Local resolution gradients and angular distribution plots were calculated in Relion and visualized in Chimera. To visualize surface electrostatic potentials, we present the coulomb electrostatic potential as implemented in Chimera. Surface hydrophobicity was colored according to the amino acid hydrophobicity scale introduced by Hessa et al.(15).

Strain generation

Yeast strains used in this study are listed in Table S3. Yeast strains carrying a deletion of either Mon1 or Ccz1 were transformed with a pRS406 plasmid carrying a GFP-tagged wild-type or mutant version of Mon1 or Ccz1, respectively, under the control of the NOP1 promotor.

Fluorescence microscopy

Microscopic analyses of yeast cells were performed as described(16). Cells were grown in yeast extract peptone medium containing glucose (YPD) overnight, diluted to OD600 of 0.25 in the morning and grown until an OD600 of around 1. Cells were collected by centrifugation (5,000 x*g*, 3 min, 20 °C) and washed in synthetic media once. For staining of the vacuole by CMAC, cells were incubated in synthetic media containing 0,1 µM CMAC for 15 min at 30 °C, washed twice in fresh media and incubated another 15 min in media without dye. Images were acquired directly afterwards using a Delta Vision Elite (GE Heathcare) equipped with an inverted microscope (model IX-71; Olympus), an UAPON X 100 (1.49 numerical aperture (NA)) oil immersion, an InsightSSI light source (Applied Precision) and a sCMOS camera (PCO). Data were processed using ImageJ 2.1.0. Shown pictures are maximum intensity projections of medial planes of yeast cells.

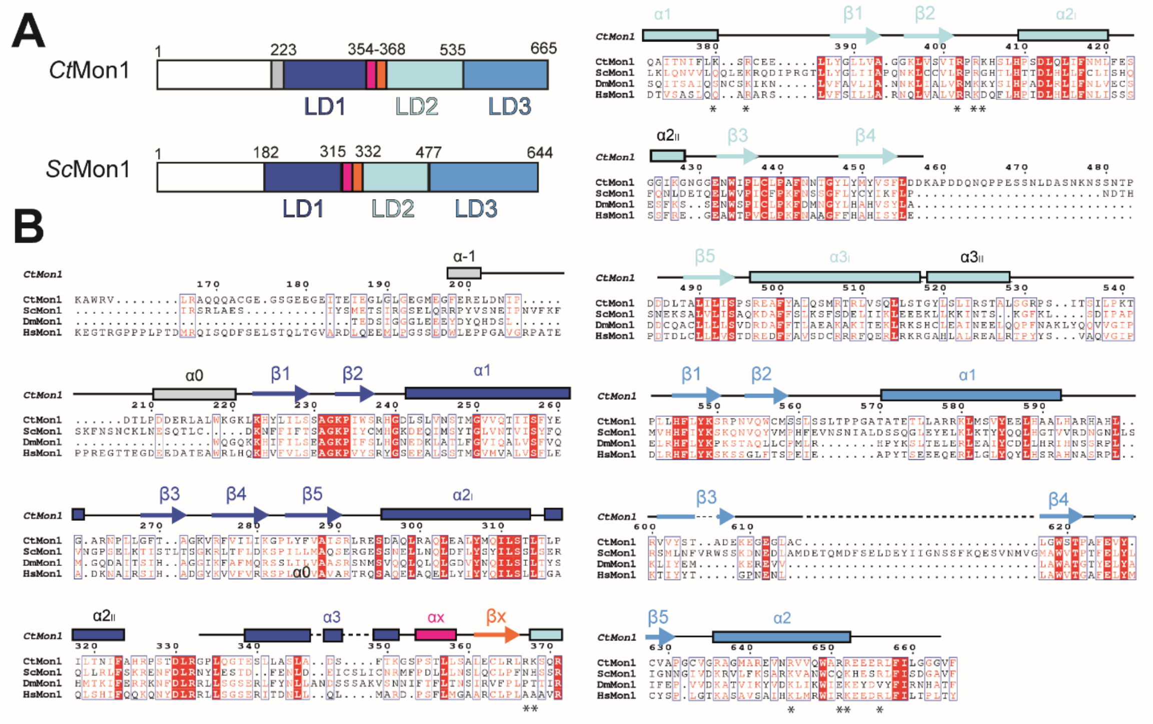

Fig. S1. Architecture of Mon1. (A) Conserved tri-longin domain arrangement of Mon1 (B) Multiple sequence alignment of Mon1 homologs with annotation of secondary structure elements. Basic residues that constitute the positive surface patch of CtMon1 are marked by asterisks.

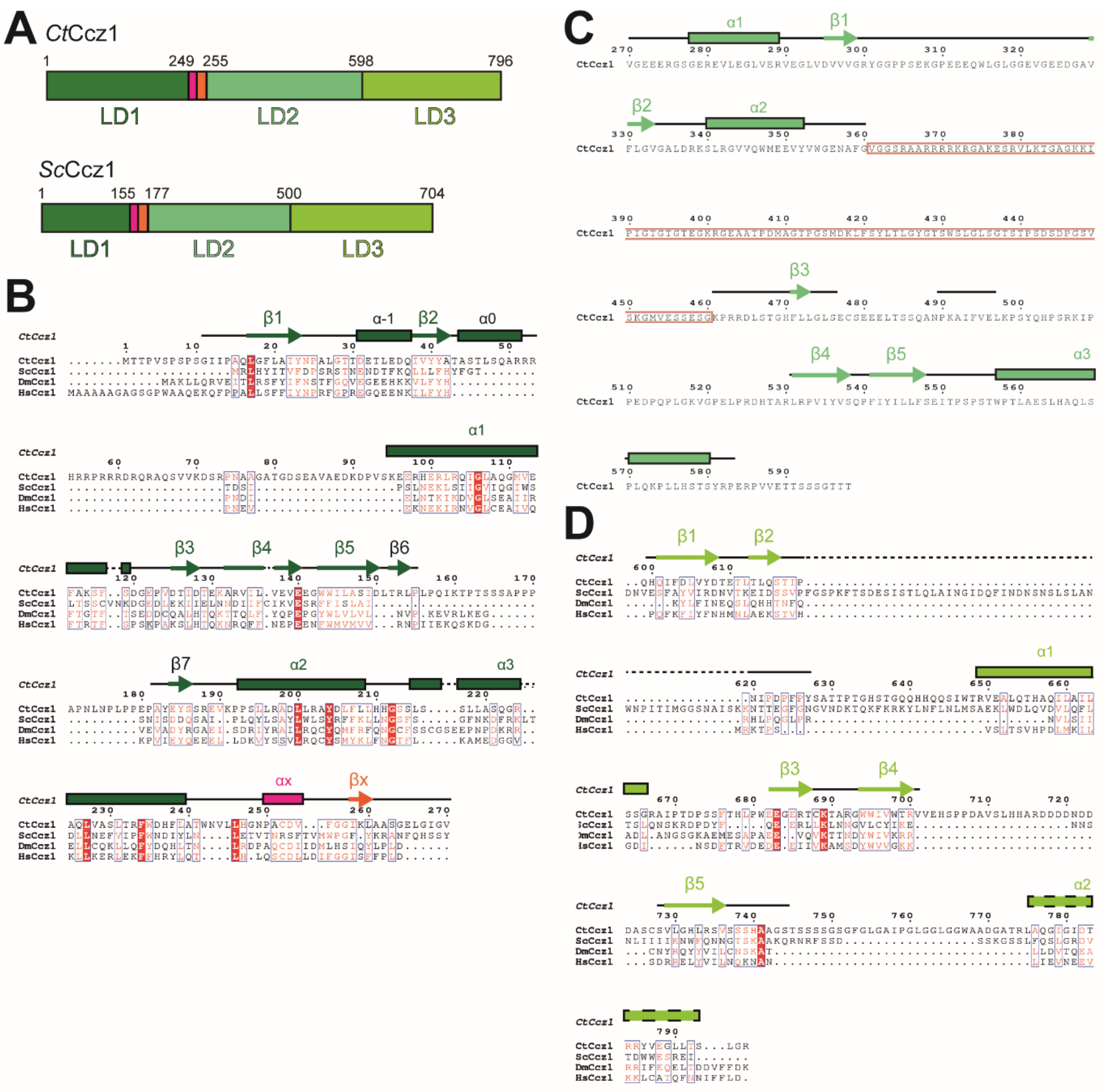

Fig. S2. Architecture of Ccz1. (A) Conserved tri-longin domain arrangement of Ccz1. (B) Multiple sequence alignment of Ccz1-LD1 with annotation of secondary structure elements. (C) Sequence of Ccz1-LD2 with annotation of secondary structure elements. The low sequence conservation and large disordered loops made a reasonable alignment with Ccz1 homologs from other species impossible. The red box marks the residues deleted in the CtCcz1ΔL construct. (D) Multiple sequence alignment of Ccz1-LD3 with annotation of secondary structure elements. The predicted helix α_2 that was not visible in the density map is annotated with a dashed box.

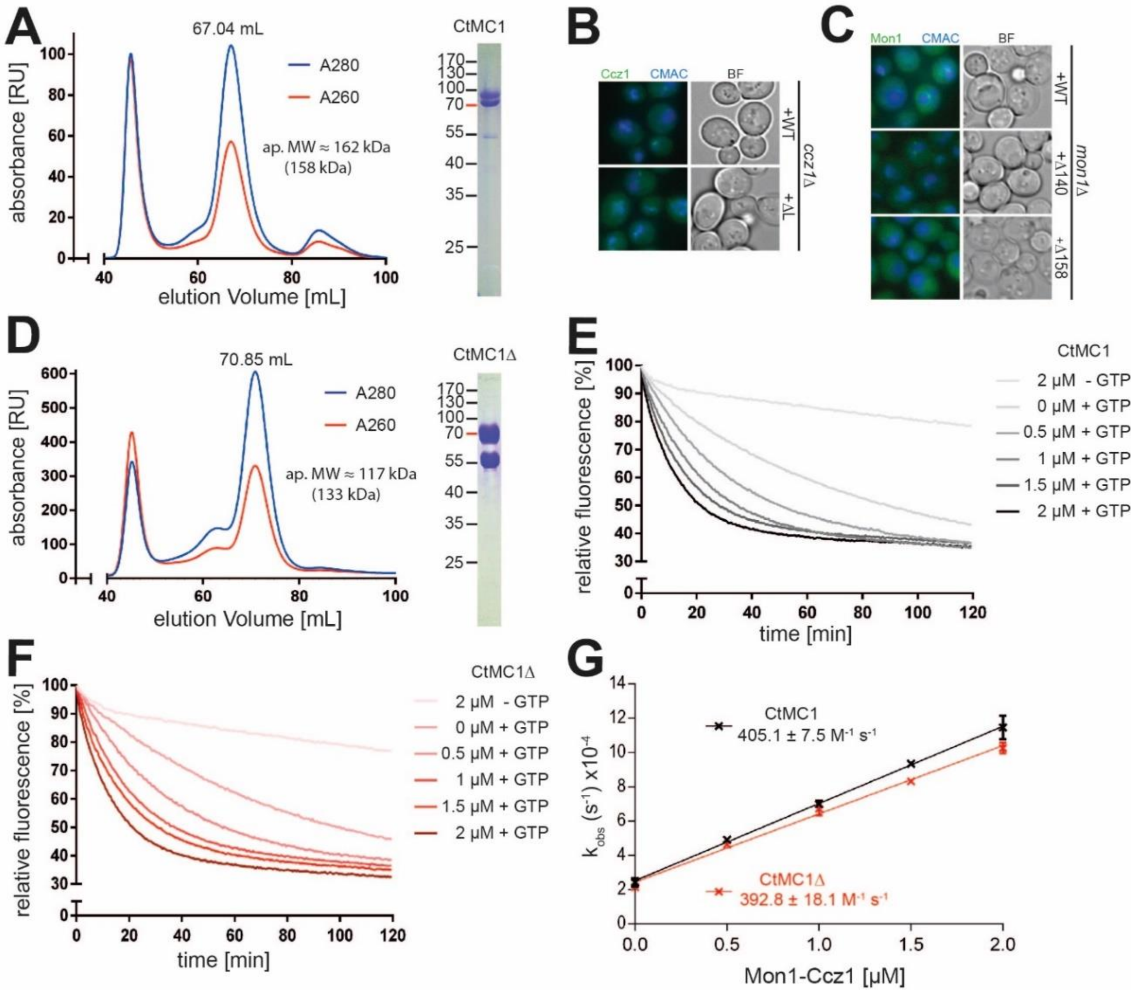

Fig. S3. Characterization of recombinant CtMC1 and CtMC1Δ. (A) Gel filtration chromatogram of the purification of CtMC1 and SDS-PAGE analysis of the pooled peak fractions. (B) GFP-tagged wild-type or truncations of scCcz1 (ΔLoop) and (C) scMon1 have been introduced into *ccz1Δ* and *mon1Δ* yeast knockout strains, respectively. Localization of GFP-tagged proteins and vacuole morphology after staining by CMAC were assessed by fluorescence microscopy. (D) Gel filtration chromatogram of the purification of CtMC1Δ and SDS-PAGE analysis of the pooled peak fractions. (E) Release of MANT-GDP from CtYpt7 was monitored in the presence of different concentrations of CtMC1 and GTP or in the absence of GTP as a control. (F) Release of MANT-GDP from CtYpt7 was monitored in the presence of different concentrations of CtMC1Δ and GTP or in the absence of GTP as a control. (G) The catalytic efficiency k_{cat}/K_M was determined by plotting CtYpt7 nucleotide exchange rates as a function of CtMC1 and CtMC1Δ concentrations, respectively. Data points represent the mean of n=3 independent biological repeats ±SD.

.

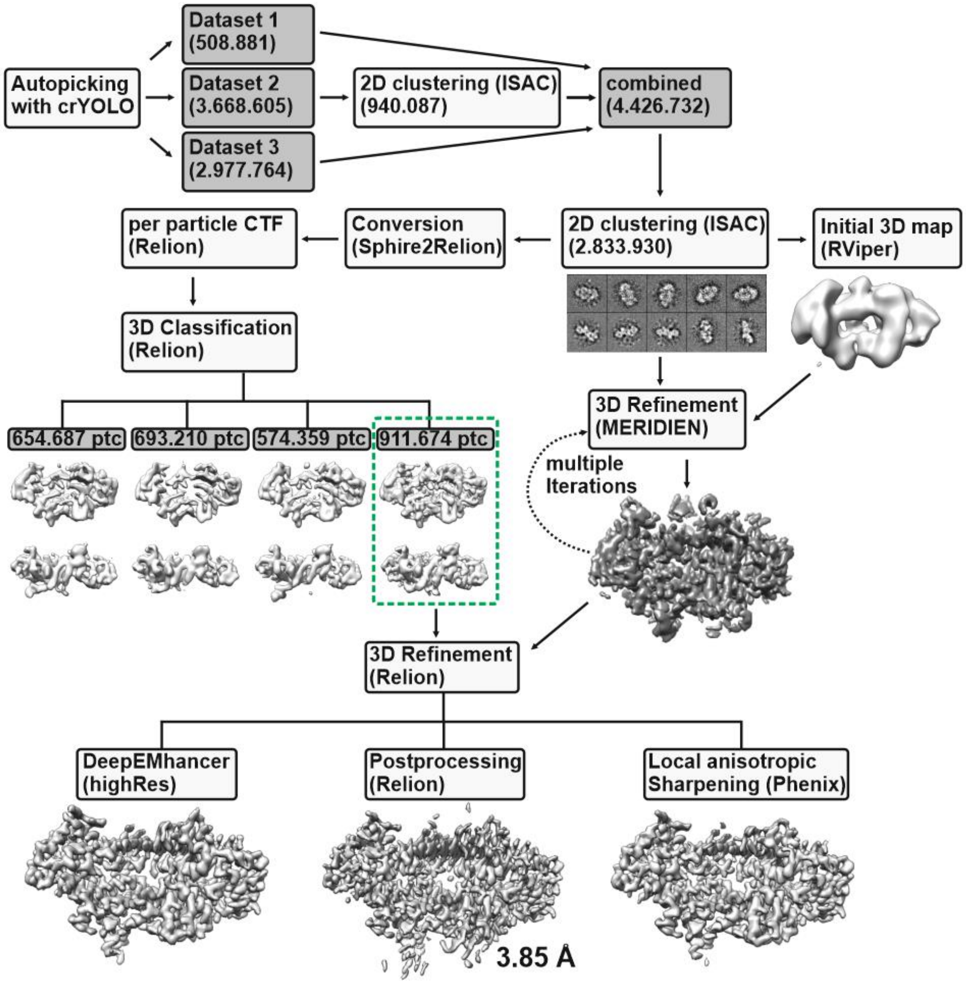

Fig. S4. Single particle processing workflow for structure determination of the Mon1-Ccz1 complex. The best class from 3D classification in Relion still contained some conformational flexibility due to multiple unresolved loops of the complex, which complicated the interpretation of some surface areas. We therefore considered 3D reconstructions obtained by three different methods for map interpretation and model building: a “classical” sharpening of the two halfmaps in Relion, a local anisotropic sharpening (which filters the map to the local resolution) in Phenix, and a deep-learning based approach by DeepEMhancer, using the implemented “highRes” training model.

.

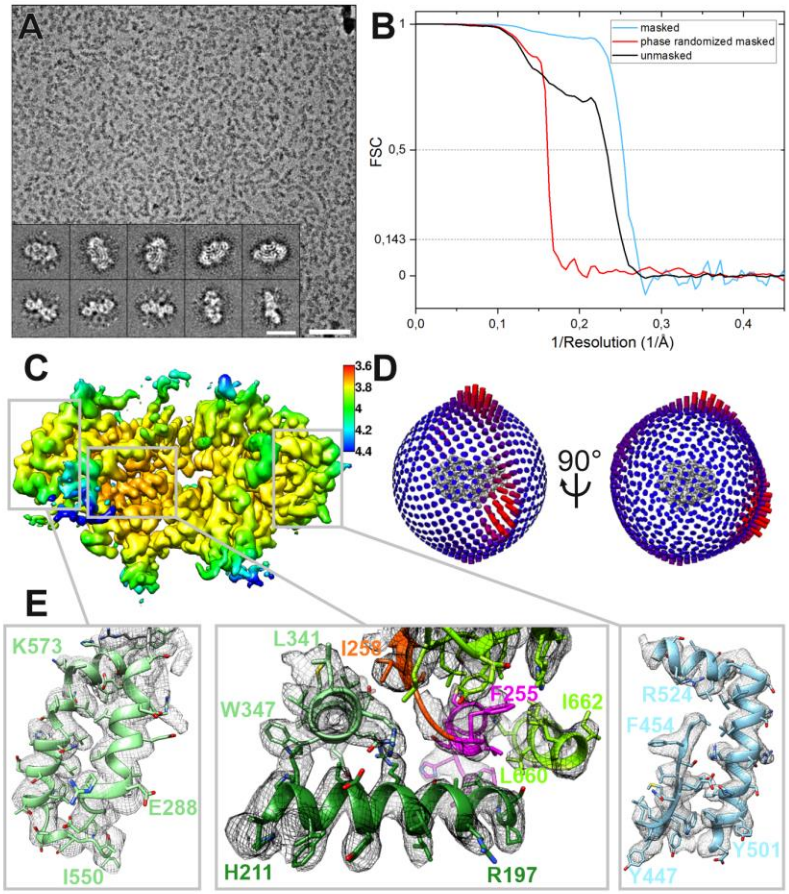

Fig. S5. Quality of the model of the Mon1-Ccz1 complex. (A) Cryo-EM micrograph and representative class averages of the Mon1-Ccz1 complex. Scale bars, 50 nm (micrograph) and 10 nm (class averages). (B) FSC of two independently refined half data sets (black line), as well as a comparison with the phase randomized data (red line). (C) Cryo-EM density map of the full dataset colored according to local resolution. (D) Angular distribution of particles that were used for the final reconstruction. (E) Examples of the molecular model and the corresponding density map from different regions of the structure.

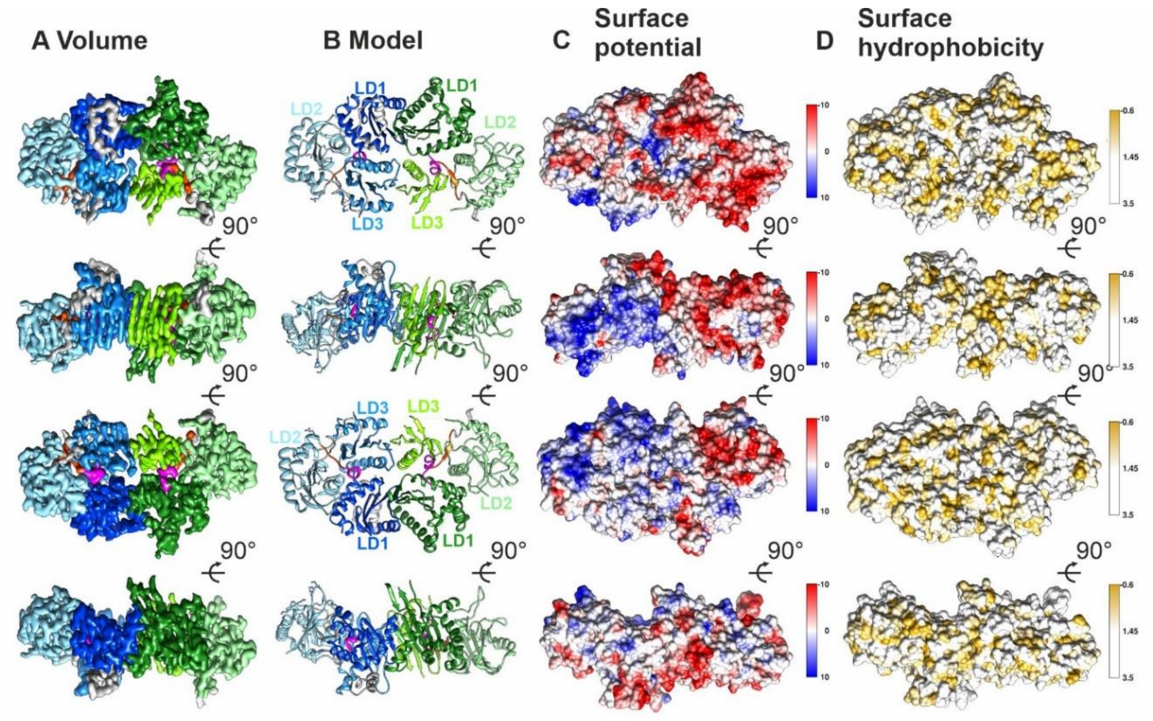

Fig. S6. Map, model and surface properties of the Mon1-Ccz1 complex. (A) 3D reconstruction of the Mon1-Ccz1 complex at 3.85 Å and (B) final model colored according to the color code as in Figure 1. (C) The Coulomb surface potential[29] and (D) surface hydrophobicity[45] derived from this.

Table S1. EM data collection and refinement statistics of the truncated CtMC1 complex. The complex was modelled into a 3D reconstruction from a substack of 911.674 particles (green box in Figure S4)

**Data collection and processing**	
Microscope	Titan Krios<br>(Volta Phase plate, XFEG)
Voltage (kV)	300
Camera	K2 summit (Gatan)
Pixel size (Å)	1.09
Number of frames	60
Total electron dose (e^-/Å^2)	72.4-73.7
Number of particles	7,155,250
Defocus range (µm)	-0.25 – -1.2
Phase Shift (degree)	20-150
Resolution (FSC@0.143, Å)	3.85
B-factor for sharpening	-218.91
**Atomic model composition**	**CtMon1ΔN, CtCcz1ΔL**
Non-hydrogen atoms	6837
Protein atoms	6837
particle substack	911.674
Ligand atoms	-
**Refinement (Phenix)**	
RMSD bond length (Å)	0.003
RMSD bond angle (°)	0.615
Model to map fit, CC mask	0.76
FSC model (Å) (0/0.143/0.5)	3.3/3.5/4.0
mean B-factor (Å^2)	96.72
**Validation**	
Clashscore	12.9
Ramachandran outliers (%)	0
Ramachandran favoured (%)	93.65
Molprobity score	2.04
EMRinger score	1.3

Table S2. Bacterial expression constructs used in this study.

Plasmid	Construct
DK65	pCDF6P-CtMon1fl<br>(GST-PreScission site CtMon1 1-665)
DK44	pCDF6P-CtMon1ΔN<br>(GST-PreScission site CtMon1 141-665)
DK123	pCDF6P-CtMon1LD1<br>(GST-PreScission site CtMon1 141-355)
DK46	pET28HS-CtCcz1fl<br>(6xHis-SUMO CtCcz1 1-796)
DK857	pET28HS CtCcz1ΔL<br>(6xHis-SUMO CtCcz1 1-360,461-796)
DK48	pET28HS-CtCcz1LD1<br>(6xHis-SUMO CtCcz1 1-249)
DK22	pCDF6P-CtYpt7fl<br>(GST-PreScission site CtYpt7 1-204)

Table S3. Yeast strains used in this study.

Strain	Genotype
CUY10471	*MATalpha leu2-3,112 ura3-52 his3-Δ200 trp-Δ901 lys2-801 suc2-Δ9 GAL natNT2::mCherry-ATG8 mon1Δ::HIS URA::pRS406-NOP1pr-GFP-MON1*
CUY10472	*MATalpha leu2-3,112 ura3-52 his3-Δ200 trp-Δ901 lys2-801 suc2-Δ9 GAL natNT2::mCherry-ATG8 mon1Δ::HIS URA::pRS406-NOP1pr-GFP-MON1Δ1-158*
CUY10478	*MATalpha leu2-3,112 ura3-52 his3-Δ200 trp-Δ901 lys2-801 suc2-Δ9 GAL natNT2::mCherry-ATG8 ccz1Δ::hphNT1 URA::pRS406-NOP1pr-GFP-CCZ1*
CUY12552	*MATalpha leu2-3,112 ura3-52 his3-Δ200 trp-Δ901 lys2-801 suc2-Δ9 GAL natNT2::mCherry-ATG8 mon1Δ::HIS URA::pRS406-NOP1pr-GFP-MON1Δ1-140*
CUY12555	*MATalpha leu2-3,112 ura3-52 his3-Δ200 trp-Δ901 lys2-801 suc2-Δ9 GAL natNT2::mCherry-ATG8 ccz1Δ::hphNT1 URA::pRS406-NOP1pr-GFP-CCZ1Δ270-403*

SI References

1. R. Danev, B. Buijsse, M. Khoshouei, J. M. Plitzko, W. Baumeister, Volta potential phase plate for in-focus phase contrast transmission electron microscopy. *Proc. Natl. Acad. Sci. U. S. A.* **111**, 15635–40 (2014).
2. S. Zheng, *et al.*, MotionCor2: anisotropic correction of beam-induced motion for improved cryo-electron microscopy. *Nat. Methods* **14** (2017).
3. A. Rohou, N. Grigorieff, CTFFIND4: Fast and accurate defocus estimation from electron micrographs. *J. Struct. Biol.* **192**, 216–21 (2015).
4. T. Moriya, *et al.*, High-resolution single particle analysis from electron cryo-microscopy images using SPHIRE. *J. Vis. Exp.* **2017** (2017).
5. T. Wagner, *et al.*, SPHIRE-crYOLO is a fast and accurate fully automated particle picker for cryo-EM. *Commun. Biol.* **2**, 218 (2019).
6. J. Zivanov, *et al.*, New tools for automated high-resolution cryo-EM structure determination in RELION-3. *Elife* **7** (2018).
7. P. D. Adams, *et al.*, PHENIX: a comprehensive Python-based system for macromolecular structure solution. *Acta Crystallogr D Biol Crystallogr* **66**, 213–221 (2010).
8. R. Sanchez-Garcia, *et al.*, DeepEMhancer: a deep learning solution for cryo-EM volume post-processing. *Commun. Biol.* **4**, 874 (2021).
9. J. Yang, *et al.*, Improved protein structure prediction using predicted interresidue orientations. *Proc. Natl. Acad. Sci. U. S. A.* **117**, 1496–1503 (2020).
10. P. Emsley, B. Lohkamp, W. G. Scott, K. Cowtan, Features and development of Coot. *Acta Crystallogr. Sect. D Biol. Crystallogr.* **66**, 486–501 (2010).
11. S. Kiontke, *et al.*, Architecture and mechanism of the late endosomal Rab7-like Ypt7 guanine nucleotide exchange factor complex Mon1-Ccz1. *Nat. Commun.* **8** (2017).
12. A. Gerondopoulos, *et al.*, Planar Cell Polarity Effector Proteins Inturned and Fuzzy Form a Rab23 GEF Complex. *Curr. Biol.* **29**, 3323-3330.e8 (2019).
13. L. Sanchez-Pulido, C. P. Ponting, Hexa-Longin domain scaffolds for inter-Rab signalling. *Bioinformatics* **36**, 990–993 (2019).
14. E. F. Pettersen, *et al.*, UCSF Chimera--a visualization system for exploratory research and analysis. *J. Comput. Chem.* **25**, 1605–12 (2004).
15. T. Hessa, *et al.*, Recognition of transmembrane helices by the endoplasmic reticulum translocon. *Nature* **433**, 377–81 (2005).
16. M. Cabrera, *et al.*, The Mon1-Ccz1 GEF activates the Rab7 GTPase Ypt7 via a longin-fold-Rab interface and association with PI3P-positive membranes. *J. Cell Sci.* **127**, 1043–51 (2014).
